# Distinct Transcriptional Signatures of Bone Marrow-Derived C57BL/6 and DBA/2 Dendritic Leucocytes Hosting Live *Leishmania amazonensis* Amastigotes

**DOI:** 10.1371/journal.pntd.0001980

**Published:** 2012-12-13

**Authors:** Emilie Giraud, Hervé Lecoeur, Guillaume Soubigou, Jean-Yves Coppée, Geneviève Milon, Eric Prina, Thierry Lang

**Affiliations:** 1 Département de Parasitologie et Mycologie, Laboratoire Immunophysiologie et Parasitisme, Institut Pasteur, Paris, France; 2 Plateforme Transcriptome et Epigénome, Institut Pasteur, Paris, France; Queensland Institute of Medical Research, Australia

## Abstract

**Background/Objectives:**

The inoculation of a low number (10^4^) of *L. amazonensis* metacyclic promastigotes into the dermis of C57BL/6 and DBA/2 mouse ear pinna results in distinct outcome as assessed by the parasite load values and ear pinna macroscopic features monitored from days 4 to 22-phase 1 and from days 22 to 80/100-phase 2. While in C57BL/6 mice, the amastigote population size was increasing progressively, in DBA/2 mice, it was rapidly controlled. This latter rapid control did not prevent intracellular amastigotes to persist in the ear pinna and in the ear-draining lymph node/ear-DLN. The objectives of the present analysis was to compare the dendritic leukocytes-dependant immune processes that could account for the distinct outcome during the phase 1, namely, *when* phagocytic dendritic leucocytes of C57BL/6 and DBA/2 mice have been subverted as live amastigotes-hosting cells.

**Methodology/Principal Findings:**

Being aware of the very low frequency of the tissues' dendritic leucocytes/DLs, bone marrow-derived C57BL/6 and DBA/2 DLs were first generated and exposed or not to live *Ds*Red2 expressing *L. amazonensis* amastigotes. Once sorted from the four bone marrow cultures, the DLs were compared by Affymetrix-based transcriptomic analyses and flow cytometry. C57BL/6 and DBA/2 DLs cells hosting live *L. amazonensis* amastigotes do display distinct transcriptional signatures and markers that could contribute to the distinct features observed in C57BL/6 versus DBA/2 ear pinna and in the ear pinna-DLNs during the first phase post *L. amazonensis* inoculation.

**Conclusions/Significance:**

The distinct features captured *in vitro* from homogenous populations of C57BL/6 and DBA/2 DLs hosting live amastigotes do offer solid resources for further comparing, *in vivo*, in biologically sound conditions, functions that range from leukocyte mobilization within the ear pinna, the distinct emigration from the ear pinna to the DLN of live amastigotes-hosting DLs, and their unique signalling functions to either naive or primed T lymphocytes.

## Author Summary

The rapid and long term establishment of parasites such as *L. amazonensis*, otherwise known to strictly rely on subversion of macrophage and dendritic leucocyte (DL) lineages, is expected to reflect stepwise processes taking place in both the skin dermis where the infective form of the parasite and the skin-draining lymph node (DLN) were inoculated. Relying on mice of two distinct inbred strains—C57BL/6 and DBA/2—that rapidly and durably display distinct phenotypes at the two sites of establishment of *L. amazonensis*, we were curious to address the following question: could live *L. amazonensis*-hosting DL display unique signatures that account for the distinct phenotypes? Based on flow cytometry, genechip and real-time quantitative PCR analyses, our results did evidence that, once subverted as cells hosting live *L. amazonensis*, DLs from C57BL/6 or DBA/2 do display distinct profiles that could account for the i) distinct parasite load profiles, ii) as well as the distinct macroscopic features of ear pinna observed once the *L. amazonensis* metacyclic promastigotes completed their four day developmental program along the amastigote morphotype.

## Introduction

*Leishmania (L.) amazonensis* perpetuates in South and Central America, its main location being the wet forests of the Amazon basin. The perpetuation of this *Leishmania* species relies successively on two hosts which cohabit more or less transiently within this ecosystem: blood-feeding sand flies and mammals, including wild rodents and humans. A broad spectrum of clinical manifestations, ranging from single cutaneous lesions to multiple, disfiguring nodules [1], [2], [3] assess the durable establisment of *L. amazonensis* as intracellular amastigotes in the dermis. As model rodents, the laboratory mice of different inbred strains can be subverted as hosts by *L. amazonensis*, the establishment of parasites in the dermis being more or less rapid. In C3H, BALB/c and C57BL/6 mice high parasite loads, coupled to non healing skin-damages are displayed at site of *L. amazonensis* inoculation and in multiple skin sites reached by parasites emigrating from the primary inoculation site [4], [5], [6], [7], [8]. By contrast, in DBA/2 mice, at the inoculation site, the *L. amazonensis* population size is rapidly controlled, a process coupled to a controlled inflammatory process with limited parasite dissemination in distant tissue(s), if any [9].

Knowing that once in the dermis of the mouse, amastigotes are hosted by mononuclear phagocytes including macrophages and dendritic leukocytes (DLs) [10], [11], [12], [13], [14], [15], we have addressed the following question: could the DLs harbouring live amastigotes contribute to the distinct phenotypes observed in C57BL/6 and DBA/2 mice? Since the frequency of DLs hosting live *Leishmania* amastigotes within the skin and skin-draining lymph nodes (DLNs) remains very low [16], [17] we decided to first conduct an *in vitro* study relying on bone marrow-derived DLs (BMD-DLs) from C57BL/6 and DBA/2 mice exposed or not to live *L. amazonensis* amastigotes.

Based on flow cytometry (FCM), genechip (Affymetrix Mouse GeneChip) and real-time quantitative PCR (RT-qPCR) analyses performed on sorted DLs hosting live *Ds*Red2-expressing *L. amazonensis* transgenic amastigotes [17] many distinct features have been highlighted. DBA/2 DLs displayed transcriptional signatures and markers that could be related to the early phenotype observed *in vivo*, in contrast to live amastigotes-hosting C57BL/6 DLs. The data are consistent with rapid and sustained immune regulatory functions accounting for the remodeling of the DBA/2 ear as *L.amazonensis* protective niche. All together this study provides, for the first time, a solid base for exploring i) the inflammatory processes that maintain the amastigote population under control in DBA/2 mice and ii) the inflammatory processes coupled to extended parasite dissemination and to poor parasite population control in C57BL/6 mice.

## Methods

### Mice

Six week old female DBA/2, C57BL/6 and Swiss *nu/nu* mice were purchased from Charles River (Saint Germain-sur-l'Arbresle, France).

### Ethics statement

All animals were housed in our A3 animal facilities in compliance with the guidelines of the A3 animal facilities at the Pasteur Institute which is a member of Committee 1 of the “Comité d'Ethique pour l'Expérimentation Animale” (CEEA) - Ile de France - Animal housing conditions and the protocols used in the work described herein were approved by the “Direction des Transports et de la Protection du Public, Sous-Direction de la Protection Sanitaire et de l'Environnement, Police Sanitaire des Animaux under number B75-15-28 in accordance with the Ethics Charter of animal experimentation that includes appropriate procedures to minimize pain and animal suffering. TL is authorized to perform experiment on vertebrate animals (licence 75-717) issued by the Paris Department of Veterinary Services, DDSV) and is responsible for all the experiments conducted personally or under his supervision as governed by the laws and regulations relating to the protection of animals.

### Preparation of *L. amazonenis* amastigotes and metacyclic promastigotes

*Ds*Red2-transgenic *L. amazonensis* strain LV79 (WHO reference number MPRO/BR/72/M1841) amastigotes were isolated from Swiss nude mice inoculated 2 months before within a BSL-2 cabinet space as described previously [17]. These amastigotes did not present any antibodies at their surface [18]. Promastigotes derived from amastigotes were cultured at 26°C in complete M199 medium. The metacyclic promastigote population (mammal-infective stage) was isolated from stationary phase cultures (6 day-old) on a Ficoll gradient.

### Monitoring of *L. amazonensis* metacylic promastigotes in ear dermis post inoculation (PI)

Ten thousand metacyclic promastigotes in 10 µl of PBS were injected into the ear dermis of C57BL/6 and DBA/2 mice. Increased ear thickness was measured using a direct reading Vernier caliper (Thomas Scientific, Swedesboro, NJ) and expressed as ear thickness.

### Preparation of BMD-DLs

DLs were differentiated from bone marrow cells of DBA/2 or C57BL/6 mice according to a method described previously [18], [19]. Briefly, bone marrow cells were seeded at 4×10^6^ cells per 100 mm diameter bacteriological grade Petri dish (Falcon, Becton Dickinson Labware, Franklin Lakes, NJ) in 10 ml of Iscove's modified Dulbecco's medium (IMDM; BioWhittaker Europe, Verviers, Belgium) supplemented with 10% heat-inactivated foetal calf serum (FCS; Dutscher, Brumath, France), 1.5% supernatant from the GM-CSF producing J558 cell line, 50 U/ml penicillin, 50 µg/ml streptomycin, 50 µM 2-mercaptoethanol and 2 mM glutamine. Cultures were incubated at 37°C in a humidified atmosphere with 5% CO_2_. On day 6, suspended cells were recovered and further cultured in complete IMDM supplemented with 10% of the primary culture supernatant before seeding on day 10 in hydrophobic 6-well plates (Greiner, St Marcel, France) at a concentration of 9×10^5^ cells/well in 3 ml complete IMDM.

### Immuno-staining for flow cytometric analyses

On day 4 post the distribution of DLs in the 6 well plate culture, DLs were exposed or not to freshly isolated *Ds*Red2-LV79 amastigotes or to live BCG at micro-organism-DL ratios of 5∶1 and 10∶1, respectively. DL cultures were placed at 34°C and sampled at 24 hours post micro-organism addition. Recovered DLs were incubated first in PBS-FCS supplemented with 10% heat-inactivated donkey serum for 15 minutes, second in PBS containing 10% FCS and 0.01% sodium azide in presence of antibodies directed against surface antigens. Extracellular staining procedures were performed with specific monoclonal antibodies (mAbs) directed against MHC class II molecules (M5/114 clone) conjugated to PE-CY5 (0.2 µg/ml) and either of the following biotinylated mAbs directed against CD86 (GL1 clone), CD80 (K-10A1 clone), CD54 (3E2 clone), CD11c (HL3) and IgG control (B81-3 clone) at 0.5 µg/ml (eBioscience,San Diego, USA). Biotinylated mAbs were revealed using 1.5 µg/ml Streptravidin conjugated to Phycoeythrin (Molecular Probes, Cergy Pontoise, France). PE-conjugated mAb directed against CXCR-4 (2B11 clone) was purchased from eBioscience. Analysis was performed on the FACSCalibur. DLs were selected on FSC-SSC parameters (to excluded debris), and on the basis of MHC class II expression to discard the fraction of “contaminating” cells expressing no surface MHC class II molecules.

Intracellular staining of amastigotes was performed after fixation in PBS containing 1% paraformaldehyde (PFA) for 20 minutes at 4°C with the 2A3-26 mAb which was shown to strictly bind to the *L. amazonensis* amastigote [18]. DLs were washed in Perm/Wash solution from the BD Cytofix/Cytoperm™ Plus Kit (BD Bioscience) and incubated with 5 µg/ml of Alexafluor 488- conjugated 2A3-26 mAb in Perm/Wash buffer for 30 minutes at 4°C in the dark. Then DLs were washed in Perm/Wash buffer and fixed with in PBS −1% paraformaldehyde (PFA).

### Immuno-staining for microscopic observations

DLs were exposed or not to freshly isolated *Ds*Red2-LV79 amastigotes at a parasite -DL ratio of 5∶1. DL cultures were placed at 34°C and sampled at 5, 24 and for 48 hours post parasite addition. Detached DLs were centrifuged on poly-L-lysine-coated glass coverslips and incubated at 34°C for 30 minutes. Cells were then fixed with 4% PFA for 20 minutes, permeabilised with saponin and incubated with 10 µg/ml of the amastigote-specific mAb 2A3-26-AlexaFluor 488 and 1 µg/ml of biotinylated-mAb (M5/114) directed against MHC class II molecules. The revelation was performed using 1.5 µg/ml streptravidin conjugated to Texas Red (Molecular Probes, Cergy Pontoise, France). Finally, they were mounted on glass slides with Hoechst 33342-containing Mowiol. Incorporation of Hoechst into DNA allowed the staining of both host cell and amastigote nuclei. Epifluorescence microscopy images were acquired on an upright microscope Zeiss Axioplan 2 monitored by the Zeiss Axiovision 4.4 software.

### Cell sorting in BSL2 containment, RNA extraction and integrity quality control

*Ds*Red2-LV79 amastigotes were added or not to cultures of C57BL/6 and DBA/2-DLs. Twenty four hours later, three samples collected from three distinct cultures of either unexposed DLs or DLs exposed to *Ds*Red2-LV79 amastigotes were carefully sorted as previously described by Lecoeur *et al.* [20]. Briefly cells were first incubated in PBS-FCS containing 0.2 µg/ml of the anti-MHC class II mAb (M5/114) conjugated to PE-Cy5-conjugated mAb (eBioscience). After two washes, cells were resuspended at 5×10^6^ cells/ml in PBS containing 3% FCS and 1% J558 supernatant. The cell sorting was performed using a FACSAria (BD Biosciences, San Jose, CA) equipped with completely sealed sample injection and sort collection chambers that operate under negative pressure. PE-Cy5 and *Ds*Red2 fluorescences were collected through 695/40 and 576/26 bandpass filters respectively. FSC and SSC were displayed on a linear scale, and used to discard cell debris with the BD FACSDiva software (BD Biosciences) [17]. *L. amazonensis* amastigote-hosting DLs were sorted by selecting cells expressing both surface MHC Class II molecules and *Ds*Red2 fluorescence and immediately collected for RNA extraction by using the RNeasy Plus Mini-Kit (Qiagen) as previously described [21]. Whatever the readout assays-Affymetrix or RT-qPCR - the RNA populations used were prepared from the same samples. The quality control (QC) and concentration of RNA were determined using the NanoDrop ND-1000 micro-spectrophotometer (Kisker, http://www.kisker-biotech.com) and the Agilent-2100 Bioanalyzer (Agilent, http://www.chem.agilent.com).

### GeneChip hybridization and data analysis

Two hundred ng of total RNA per sample were processed, labelled and hybridized to Affymetrix Mouse Gene ST 1.0 arrays, following Affymetrix Protocol (http://www.affymetrix.com/support/downloads/manuals/expression_analysis_technical_manual.pdf). Three Biological replicates per condition were run. Following hybridization, the arrays were stained and scanned at 532 nm using an Affymetrix GeneChip Scanner 3000 which generates individual CEL files for each array. Gene-level expression values were derived from the CEL file probe-level hybridization intensities using the model-based Robust Multichip Average algorithm (RMA) [22]. RMA performs normalization, background correction and data summarization. An analysis is performed using the LPE test [23](to identify significant differences in gene expression between parasite-free and parasite-harbouring DLs, and a p-value threshold of p<0.05 is used as the criterion for significant differential expression. The estimated false discovery rate (FDR) was calculated using the Benjamini and Hochberg approach [24] in order to correct for multiple comparisons. A total of 1,340 probe-sets showing significant differential expression were input into Ingenuity Pathway Analysis software v5.5.1 (http://www.ingenuity.com), to perform a biological interaction network analysis. The symbols of the modulated genes are specified in the text (fold change [FC] values between brackets), while their full names are given in additional file 1. MIAME-compliant data are available through GEO database http://www.ncbi.nlm.nih.gov/geo/ accession GSE

### Validation of microarray analyses by RT-qPCR

Total RNAs from DLs cultures were reverse-transcribed to first strand cDNA using random hexamers (Roche Diagnostics) and Moloney Murine Leukemia Virus Reverse Transcriptase (Invitrogen, Life Technologies). A SYBR Green-based real-time PCR assay (QuantiTect SYBR Green Kit, Qiagen) for relative quantification of mouse target genes was performed on a 384-well plate LightCycler 480 system (Roche Diagnostics). Crossing Point values (Cp) were determined by the second derivative maximum method of the LightCycler 480 Basic Software. Raw Cp values were used as input for qBase, a flexible and open source program for qPCR data management and analysis [25]. Relative expression for 8 transcripts (*ccl2, cl17, ccl19, ccr1, ccr2, cxcr4, cd274, tnfsf4*) were calculated for sorted LV79-hosting DLs using sorted DLs from *Leishmania* unexposed cultures as calibrators. For normalization calculations, candidate control genes were tested *(pgk1, h6pd, ldha, nono, g6pd, hprt, tbp, l19, gapdh, rpIIe* and *ywhaz)* with the geNorm [26] and Normfinder programs [27]. *Tbp* and *nono* were selected as the most stable reference genes for the C57Bl/6 DLs. *RpIIe* and *tbp* were selected for the DBA/2 DLs.

### Transcriptional analysis in tissues by RT-qPCR

At day 4 and 7 post the inoculation of 10^4^ metacyclic promastigotes, three mice were sacrificed, the abundance of some transcripts being determined by real time RT-qPCR. Control, naïve mice were analyzed in parallel. Whole ear pinnas and ears-DLN were removed and fragmented using the Precellys 24 System [21]. Total RNAs were extracted and processed for RT-qPCR as described above. *Ldha* and *nono* were selected as the most stable reference genes for the C57Bl/6 and DBA/2 ears. *tbp* and *nono* were selected for the as the most stable reference genes for C57Bl/6 DLNs while *ywhaz* and *nono* were selected for the DBA/2-DLNs.

### *Leishmania* quantification in ears and ears-DLN

The experimental procedure for quantifying *Leishmania* in tissues was done as previously described by de La Llave et *al* [21]. Briefly, serial 10-fold dilutions of parasites (from 10^8^ to 10^1^) were added to either ears or ear-DLN recovered from C57BL/6 or DBA/2 naive mice. Total RNAs were extracted and processed for RT-qPCR as described above. The primers for *Leishmania* gene target (*ssrRNA*) to quantify the number of parasites were F- CCATGTCGGATTTGGT and R- CGAAACGGTAGCCTAGAG [28]. A linear regression for each standard curve was determined: number of parasites against the relative expression of *ssrRNA* values.

### Statistical analyses

Two-sided Student's paired t-tests were used to compare FCM experiments (4<n<6). A Mann-Whitney test was used to compare ear thickness measurements and number of parasites.

## Results and Discussion

### *L. amazonensis* amastigotes set up in distinct dermis niche post inoculation in C57BL/6 and DBA/2 mice

**Figure 1:**
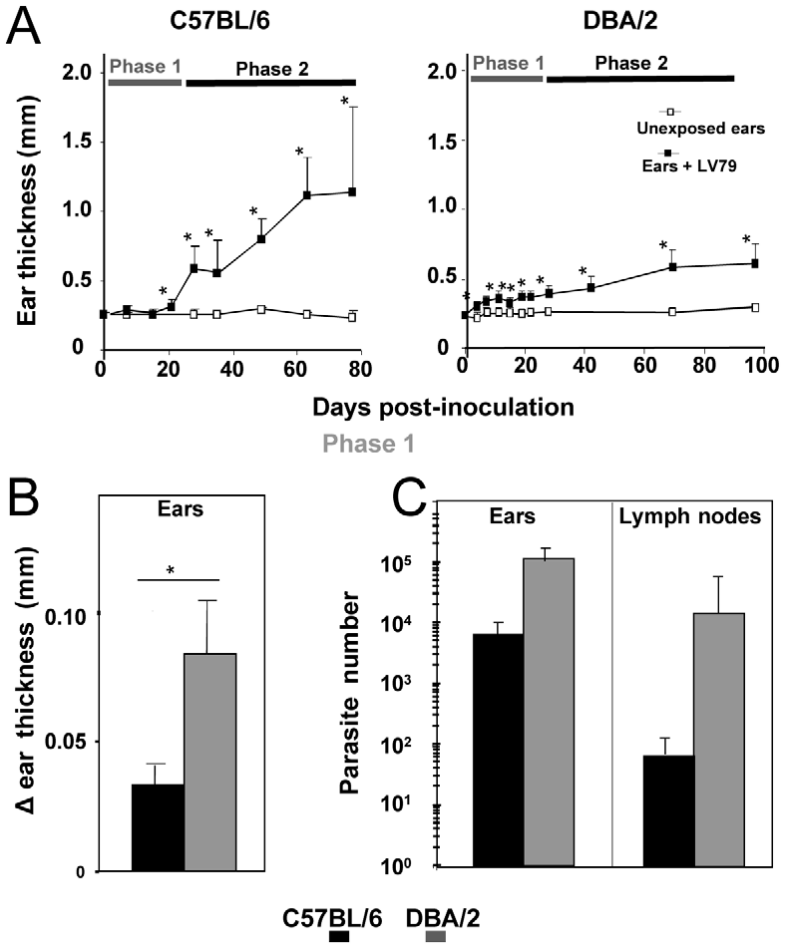
Distinct ear pinna features and amastigote population size values in C57BL6 and DBA/2 mice. Ten thousand LV79 metacyclic promastigotes were inoculated into the ear dermis of C57BL/6 (n = 41) and DBA/2 (n = 65) mice. **A–C**) Ear thickness was monitored post LV79 inoculation (black dots) or not (white dots) in ears up to 80 days for C57BL/6 mice and up to 100 days for DBA/2 mice. Results are expressed as means and standard deviations. * indicates significant p values between inoculated and un-exposed/control ears. Phase 1 (day 0 to day 22 PI) and phase 2 (post day 22) are depicted. **B, C**) Mean delta of ear thickness and parasite load in C57BL/6 and DBA/2 mice are displayed over the phase 1 (day 4 to day 22). (B) – The delta of ear thickness was obtained from inoculated ear thickness measures of all mice from day 4 to day 22 minus ear thickness values of unexposed/control mice - (**C**) Mean amastigote load quantification in ears and ears-DLNs of C57BL/6 and DBA/2 mice by RT-qPCR (n = 12 mice) over the phase 1. Means and SDs are shown. * p<0.002.

C57BL/6 and DBA/2 mice were given into the ear pinna dermis a low number (10^4^) of *L. amazonensis* (LV79 strain) metacyclic promastigotes. The monitoring of ear macroscopic features up to 100 days post inoculation (PI) has evidenced mouse inbred strain-specific features (Figure 1). C57BL/6 mice did not display any significant inflammatory signs during the early phase (ranging from day 0 to day 22 PI, phase 1), whereas they later display sustained inflammatory signs (after 22 days, phase 2; figures 1A, 1B). During the early phase, only a few parasites can be quantified in the ear pinna, the ear pinna-DLN displaying lower number of parasites (<100 parasites/DLN; figure 1C). In contrast, in DBA/2 mouse ear pinna, a mild inflammatory process was observed immediately post the inoculation whereas a rapid increase of the amastigote population size was noted in both the ears and ears-DLN. The second phase was delineated by the persistence of inflammatory process (Figure 1) coupled to the control of parasite load in the ear pinna and ear-DLN (data not shown).

We reasoned that early distinct DLs-dependent immune processes- promoting either rapid or slow remodeling of the dermis as amastigote-protective niches- could account for the distinct features displayed, over time, by the *L. amazonensis* amastigotes-hosting ear pinna of the C57BL/6 and DBA/2 mice. Being aware that, whatever the tissues, the DL frequency is very low, we considered biologically sound to start the comparative analysis with GM-CSF-dependent C57BL/6 or DBA/2 cultured DLs, once they were hosting, or not, live *L. amazonensis* amastigotes. Briefly, C57BL/6 and DBA/2 bone marrow cell suspensions were exposed or not to live DsRed2 *L. amazonensis* amastigotes and carefully sorted from otherwise heterogeneous cultures. The immunolabelling of surface MHC class II allowed us to exclude the low fraction of amastigote-hosting cells that did not express surface MHC class II. The subsequent step of such an approach was to first monitor, at the transcriptional level with the Affymetrix-based technology any potential distinct reprogramming of live *L. amazonensis* amastigotes-hosting DLs.

### A reliable *in vitro* model for comparing features of C57BL/6 and DBA/2 DLs harbouring *L. amazonensis* amastigotes

**Figure 2:**
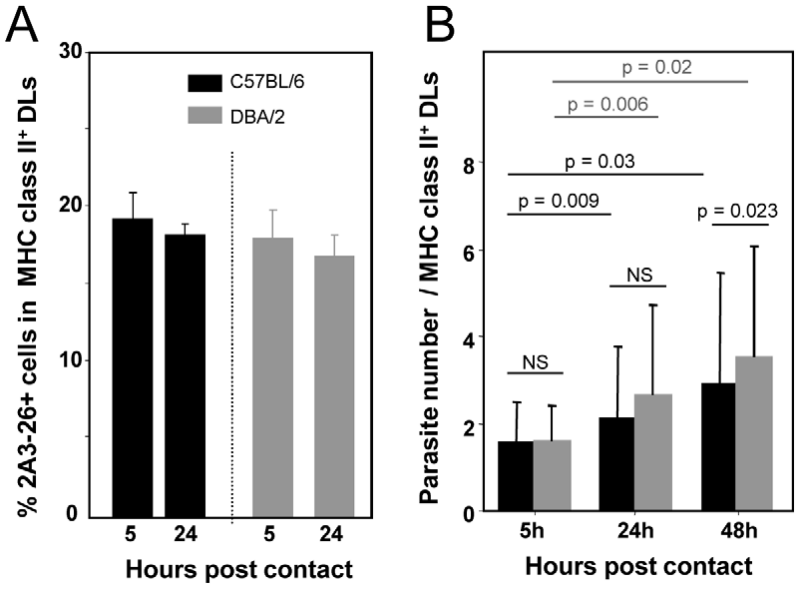
Monitoring of amastigotes-loaded C57BL/6 and DBA/2 DLs. **A, B**) LV79 amastigotes were added to BMD-DL cultures (MOI of 5∶1) and sampled at 5, 24 and 48 hours. Fluorescence microscopy analyses were performed exclusively on MHC class II positive (High and Low) DLs. The intracellular amastigotes were evidenced by the 2A3-26 staining of fixed and permeabilized DLs from C57BL6 and DBA/2 mice (black and grey histograms, respectively). (**A**) Percentages of 2A3-26^+^ cells and (**B**) number of intracellular amastigotes in BMD-DLs from C57BL/6 or DBA/2 mice. Means and SDs are shown. Statistical differences obtained between mouse genotypes and time points are indicated.

We used a carefully designed *in vitro* model [20] based on cultures of mouse BMD-DLs in which more than 97% of cells expressed CD11c, CD11a and CD11b (data not shown). When the presence/absence of surface MHC class II molecules was monitored on whole cell cultures by fluorescence microscopy and FCM, three phenotypically distinct cell subsets were evidenced (Figures S1A–C). The population of cells that did not express surface and intracellular MHC class II molecules were considered as “Contaminating” Cells (CC). The two other cell populations partition between i) a majority of cells displaying a moderate surface MHC class II amount (MHC II^low^; *bona fide* immature DLs) and ii) a minority of cells expressing very high levels of MHC II molecules (MHC II^high^; *bona fide* mature DLs). *Ds*Red2 *L. amazonensis/LV79* amastigotes were put in contact with BMD-DLs (MOI of 5/1) and analysed 5, 24 or 48 hours later (Figure 2). Intracellular amastigotes (2A3-26^+^) detected by immunofluorescence microscopy analysis were evidenced in all BMD-DL subsets with much higher number of amastigotes in CC (data not shown). Low percentages of DLs hosting 2A3-26^+^ parasites were also documented by FCM analyses at 24 hours post amastigote addition (23.0%+/−12.6 and 26.0%+/−8.1 of 2A3-26^+^ cells in C57BL/6 and DBA/2 BMD-DLs, respectively, for n = 9 experiments). Interestingly, while the percentage of DLs housing amastigotes did not change from 5 hours to 24 hours (Figure 2A), the number of intracellular amastigotes did slowly expand whatever the mouse genotype (Figure 2B) over the otherwise limited temporal window we did focus on. *L. amazonensis* amastigote-hosting DLs were sorted by selecting cells expressing both surface MHC Class II molecules and *Ds*Red2 fluorescence (see below).

### Distinct signatures displayed by sorted C57BL/6 and DBA/2 DLs housing live *L. amazonensis* amastigotes

#### Gene expression profiles of DLs hosting or not live amastigotes

We next performed a genome-wide transcriptional analysis by comparing the gene expression profiles of control DLs unexposed to *Leishmania* amastigotes and live amastigote-hosting DLs from both mouse strains. To provide a robust comparative analysis and allow generating a detailed picture of the distinct features of C57BL/6 and DBA/2 DLs, a special attention was given to specifically gated or sorted cells expressing class II molecules (Figures S1A, S1C; subsets MHC II^low^ and MHC II^high^).

**Figure 3:**
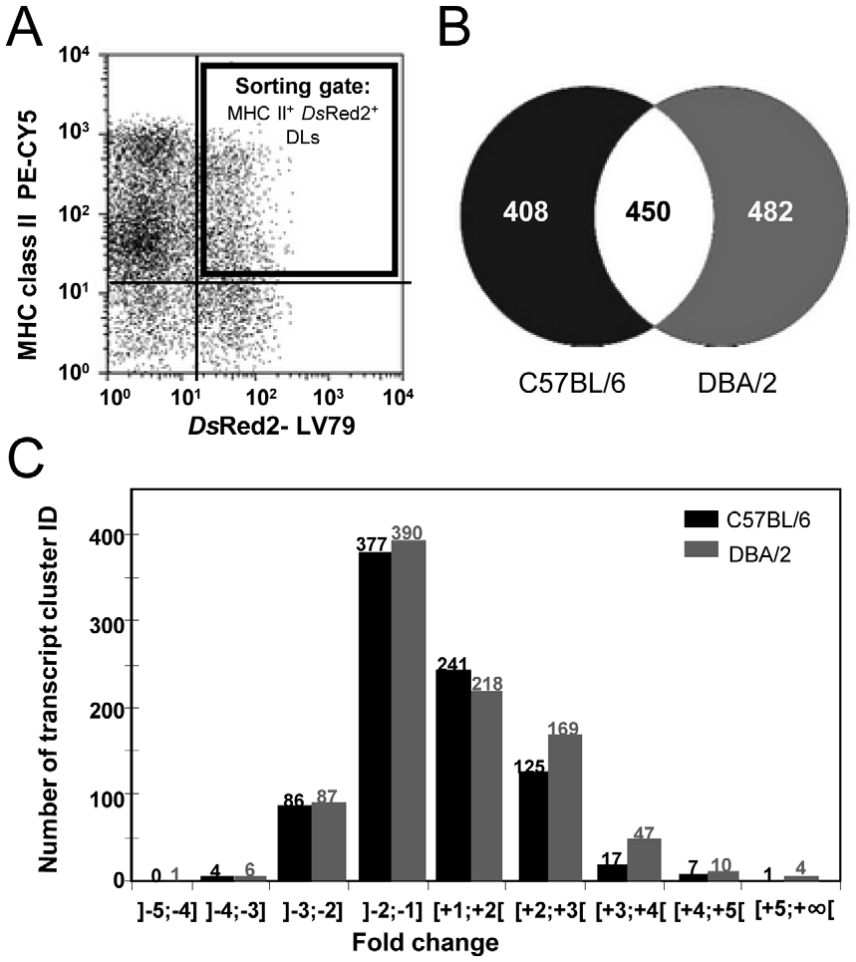
Transcriptional analysis of DLs hosting LV79 amastigotes. C57BL/6 and DBA/2 BMD-DL cultures were incubated or not with *Ds*Red2-LV79 amastigotes (MOI of 5∶1) for 24 hours. **A**) Gating strategy for sorting the DLs that were hosting live amastigotes. The double labelling of i) surface MHC class II molecules-detected by an antibody conjugated to PE-CY5- and ii) intracellular transgenic LV79 expressing the fluorescent *Ds*Red2 allowed the sorting of DLs hosting live amastigotes (black gate). **B, C**) Global analysis of modulated transcripts in C57BL/6 and DBA/2 DLs hosting live LV79 amastigotes. Once control DLs and amastigote-hosting DLs were sorted their respective total RNA were prepared and further processed for Affymetrix-based analyses. (**B**) Representation of the number of specific and common transcripts modulated in live amastigotes-hosting DLs sorted from C57BL/6 and DBA/2 bone marrow cultures. (**C**) Histogram showing the number of modulated transcripts according to their fold change values.

Despite their low frequency, MHC positive DLs hosting live parasites were easily sorted by using the detection of fluorescent *Ds*Red2 protein expressed in transgenic *L. amazonensis* amastigotes [17] (Figure 3A). Affymetrix analyses performed on total RNAs revealed that sorted DLs hosting *Ds*Red2 LV79 amastigotes displayed discrete transcriptional modifications compared to control BMD-DLs, both in term of modulated gene numbers and of modulation magnitude. Out of 28,853 mouse genes, 858 and 932 were captured with a differential expression at the 5% significance level in C57BL/6 and DBA/2 DLs, respectively (Figure 3B). Of these, 450 were common between the two mouse strains (Figure 3B). A similar fold-change distribution was observed whatever the DL genetic background (Figure 3C). We first evidenced common transcripts of C57BL/6 and DBA/2 DLs such as those involved in arginine metabolism which could be play an important role in the early parasite multiplication within DLs from both strains (Figure S2). Indeed, transcripts for key enzymes involved in pathway of arginine metabolism such as Arginase-1 (*arg1*), monoamine oxydase A (*maoa*) and spermidine/spermine N1-acetyltransferase 1 (*sat1*) were up regulated (Figure S2). These modulations were also confirmed by the gold standard approach of RT-qPCR (data not shown). Consequently, *L. amazonensis* amastigotes probably sustain a metabolic flux that promotes the biosynthesis of polyamines to facilitate the intracellular amastigote multiplication which was evidenced as soon as 24 hours post-addition of amastigotes (Figure 2B).

These analyses being set up, we found biologically relevant to focus on distinct transcriptional signatures. Such a choice allows highlighting some components of the early immune shaping of C57BL/6 versus DBA/2 ear pinna as unique skin sites where the amastigote population size increase is respectively slow or rapid. Curiously, while a delayed amastigote growth profile is coupled to clinically silent process in the C57BL/6 ear pinna, the rapid amastigote growth onset in the DBA/2 mouse ear pinna is coupled to a mild inflammatory process.

#### Distinct transcriptional signatures of C57BL/6 and DBA/2 DLs hosting live amastigotes

We focused our attention on two different-though temporally linked families- of transcriptional signatures that were unique to each mouse: i) transcripts related to DL adhesion and de-adhesion, the expected outputs being distinct local live amastigote-hosting DL dissemination within the dermis and distinct emigration of live amastigote-hosting DL from the ear to the ear-DLN and ii) transcripts linked to the interactions between DLs and non DL leucocytes, a special attention being given to T lymphocytes which are known to be the main partners of DLs in the LNs draining any peripheral upstream tissues.

#### Signatures that could contribute to distinct leucocyte mobilization within the ear pinna

The mild inflammation displayed from day 4 onward post metacyclic promastigotes in DBA/2 ears is likely assessing complex processes. Affymetrix-based analyses revealed complex patterns of transcriptional modulations of pro-inflammatory chemokines and chemokine receptors that can promote the recruitment and/or persistence of different leucocyte lineages in the dermis, contributing to the rapid amastigote establishment and expansion (Figure S3). These transcript modulations in DBA/2 DLs included i) *cxcl4* (+2.88) for the maintenance of senescent neutrophils and the recruitment of monocytes [29], ii) *ccl2* (+3.38; RT-qPCR:+22.2), *ccl3* (+4.58), *ccl4* (+2.58), *ccl7* (+2.57) and *ccl19* (+1.16; RT-qPCR:+6.8) [30] for the recruitment of NK lymphocytes, and iii) *ccl2* (+3.38) [31], [32] for the recruitment of monocytes/macrophages [31], [32]. By contrast, we evidenced a weak modulation of these transcripts in C57BL/6 DLs which could be related to the absence of inflammatory process during the early phase in mice.

These variations may be associated to modulation of transcripts which encode for molecules involved in the invasive properties of DLs hosting live amastigotes within the skin. The dermal extracellular matrix (ECM) features are among the contributors to the DL invasiveness. Different metallo-proteinases known to act on the dermis ECM proteins could locally remodel the ECM facilitating DL migration though the remodelled ECM. Thus, among the transcript data sets, a particular attention was given to transcripts coding the Matrix Metallo Proteinases/MMPs. In DBA/2 DLs hosting live amastigotes, transcripts encoding for MMP12 (+1.45) and MMP2 (+2.53) were specifically increased. MMP12 has potent ECM remodelling properties due to its specific elastolytic activity, but may also participate to the inflammatory process through the activation of TNF [33]. Moreover, MMP12 presents potent direct pro-inflammatory properties including the ability to induce neutrophil influx, cytokine and chemokine production [34]. Finally MMP2 was also shown to activate the IL-1 precursor to the active form [35], [36]. Altogether, these DBA/2-specific transcriptional modifications could favour a local but mild inflammation that could be critical for the further development of an effective immunological control of *Leishmania* amastigote population size. By contrast, in C57BL/6 DL-hosting live amastigotes, despite the up regulation of *ccl2* (+1.90; RTq-PCR +6.34) and *ccl3* transcripts (+3.61), the concomitant down modulation of transcripts coding for inflammatory cytokine receptors IL-1R2 (−1.94), Il-1Rl1 (−2.38) and IL2Rα (−1.99) and the up regulation of IL1rn (+1.69) may account for the absence of clinically detectable inflammatory process during the phase 1.

#### Distinct transcriptional signatures that could contribute to a distinct emigration from the ear pinna to the DLN of live amastigotes-hosting DLs

**Figure 4:**
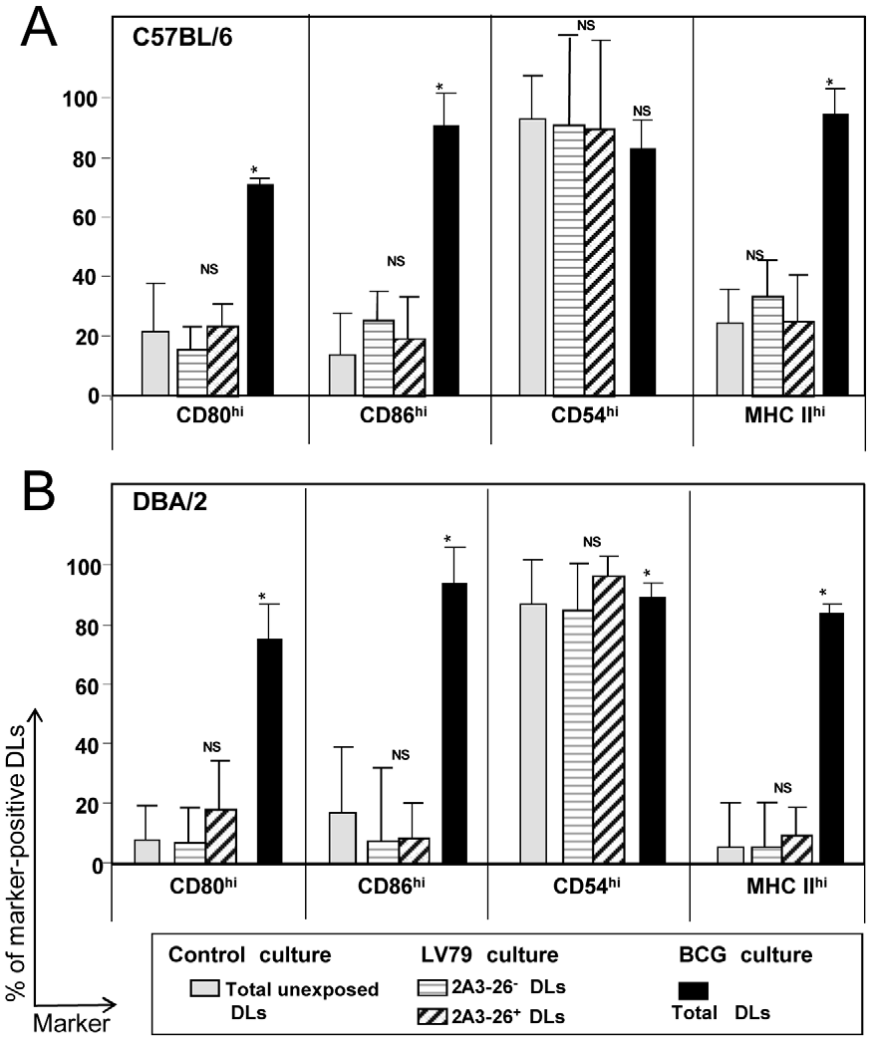
Evaluation of the maturation features of DLs loaded with LV79 amastigotes. C57Bl/6 and DBA/2 BMD-DL cultures were incubated or not with LV79 amastigotes (MOI of 5∶1) or BCG (MOI of 10∶1) for 24 hours. Control cultures without exposition to microorganisms (unexposed DLs), LV79-exposed DL cultures and BCG-exposed DL cultures were analyzed by FCM. Histograms of gated C57BL/6 (A) or DBA/2 (B) BMD-DLs expressing surface MHC class II molecules are shown. The percentage of DLs expressing high cell surface levels of CD80, CD86, CD54, MHC class II are shown as means and standard deviations (4≤n≤7 independent experiments). In LV79-exposed DL cultures, the distinction between parasite-free (2A3-26- DL) and amastigote-hosting DLs (2A3-26^+^ DLs) was based on the recourse to the 2A3-26 mAb which binds exclusively to *L. amazonensis* amastigotes. * indicates a significant difference between control cultures and microorganisms-harbouring BMD-DLs (p<0.001).

The modulation of transcripts encoding for so called “classical maturation markers” such as CD54 and CD80 and MHC class II molecules was not observed in DBA/2 and C57BL/6 DLs post contact with living amastigotes. This state of “immature-like phenotype” of DLs has been confirmed by the absence of surface expression modulation of these classical maturation markers in live amastigotes-hosting DLs. This apparent lack of maturation process strongly contrasted with the “full maturation” of DLs exposed to live BCG (Figures 4A, 4B). Of note, the absence of modulation of these markers has been also evidenced in the 2A3-26^+^ (amastigote loaded) versus 2A3-26^−^ (amastigote-free) DLs present in the same culture (Figures 4C, 4D).

**Figure 5:**
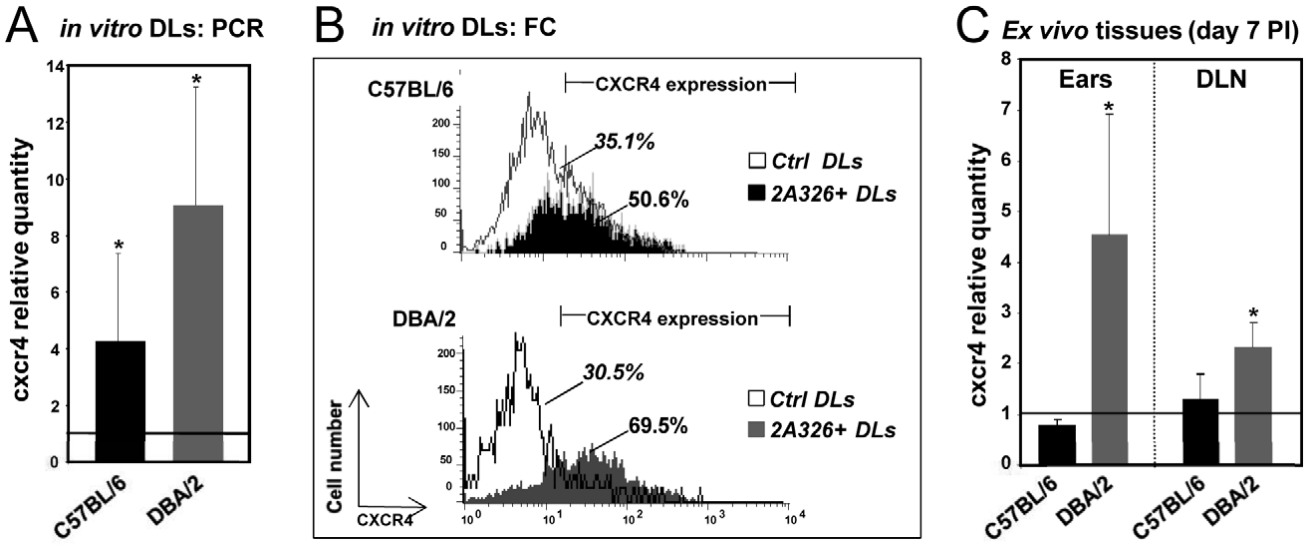
Validation of microarray analyses. **A**) RT-qPCR analysis of *cxcr4* transcript modulation in sorted BMD-DLs hosting live *L. amazonensis* amastigotes, the BMD-DLs unexposed to amastigotes being the calibrators for each mouse genotype. Means and standard deviations are shown (n = 3 independent experiments). Significant *p* values are indicated *p<0.05. **B**) FCM analysis of CXCR4 expression. Intracellular amastigotes were evidenced by the intracellular immunostaining with 2A3-26 mAbs conjugated to Alexa Fluor (green fluorescence) and CXCR4 expression by PE-conjugated anti-CXCR4 antibodies. CXCR4 expression was analyzed in unexposed DLs to *Leishmania* (Ctrl DLs; white histograms) and in 2A3-26^+^ DLs from DL cultures exposed to *Leishmania* amastigotes (black and grey histograms represent DLs from C57BL/6 and DBA/2 mouse genotypes, respectively). One representative experiment of 4 independent experiments is shown. **C**) RT-qPCR analysis of *cxcr4* abundance in ears and ear-DLNs of *L. amazonensis*-hosting C57BL/6 and DBA/2 mice. Three mice of each species were inoculated with 10^4^ *L. amazonensis/*LV79 metacyclic promastigotes into the ear dermis. For control, three mock mice were used as calibrator for calculating relative expressions. At day 7 PI, ears and DLNs RNA were extracted and reverse transcribed and the real time PCR assay was performed as described in Methods. Displayed data are the mean fold changes for cxcr4 transcripts. Significant values between control and *L. amazonensis*-hosting mice are indicated *p<0.05. One representative experiment of 3 independent experiments is shown.

However, we noticed transcriptional signatures of alternative DL maturation pathways: for instance, in DBA/2 DLs hosting live amastigotes, we evidenced a strong increase in transcripts coding for CXCR4 molecules (+2.47; RT-qPCR+9.05) which may drive the “maturing” DLs first towards the lymphatic vascular bed and then into T cell areas within the lymph nodes (supporting data S3). This observation was confirmed by RT-qPCR (Figure 5A) and importantly also at the protein level by FACS analysis (Figure 5B). Moreover an up regulation of *ccl17* (+1.88; RT-qPCR 1.34) transcripts was observed in DBA/2 DLs that could account DL sensitization to CXCR4-dependent migration from the skin to DLNs [37]. Interestingly, an up-regulation of *cxcr4* transcripts was noted in DBA/2 but not in C57BL/6 ears and ear-DLNs sampled at day 4 and 7 post *L. amazonensis* inoculation (Figure S3; figure 5C). We also evidenced in DBA/2 BMD-DLs a down modulation of transcript coding for CCR1 (−1.68; RT-qPCR −2.13) which was shown to be displayed on immature DLs and down modulated at the initiation of the maturation process [38]. In addition, as already mentioned, *ccl9* (+2.13), *ccl2* (+3.38), *ccl19* (+1.16; RT-qPCR 6.8) transcripts were up regulated in DBA/2 DLs: the higher abundance of ccl19 transcripts might probably result from an increased CCL2 production (+3.38; RT-qPCR +22.2) and CCL2 ligation to its receptor (CCR2) [39]. Moreover, in addition to the up-regulation of as *mmp2* (+2.53) [40] we evidenced the up regulation of transcripts encoding for CXCL14 (+2.97) a potent chemo-attractant and activator of DLs which might be involved in DL homing *in vivo* [41], [42]. We also evidenced other transcripts such as *cdc42ep* (+1.62) coding for the CDC42EP2 which acts downstream of the Rho GTPase CDC42 to induce pseudopodia formation and play a decisive role in DL migration *in vivo* through adequate coordination of both cortical and non-cortical cytoskeletal flow [43]. Additionally we noted an increase of transcripts encoding for the GTPase activating protein ArhGAP10 (+2.15) which functions as GTPase-activating protein for Cdc42 and F-actin dynamics at the level of Golgi apparatus [44]. All together these results seem to indicate a modulation of components that sequentially elicit key changes in dynamics and in the architecture of cells, allowing coupled migration and maturation of DBA/2 live amastigotes-hosting DLs.

By contrast, we evidenced a modulation of small number of transcripts in C57BL/6 BMD-DLs. Thus, *ccr2* transcript was down regulated (−1.68; RT-qPCR : −2.08), without any change in *ccl19* transcript abundance, suggesting that no maturation could be initiated in those live amastigote-hosting DLs. We also noted a significant down modulation of transcripts encoding DC-SIGN (*cd209c*; −2.21), a lectin otherwise known to be involved in DL-neutrophil cross-talks, that could prevent the Mac-1 and CEACAM1-displaying neutrophil-dependant DL maturation [45]. Moreover, DC-SIGN is also involved in the specific migratory processes of DLs, through the binding to ICAM-2 which is abundantly expressed by blood and lymphatic vascular bed [46].

Thus, all together these *in vitro* generated data highlight transcriptional signatures that may contribute to the distinct features observed in C57BL/6 versus DBA/2 ear pinna and in the ear pinna-DLNs during the first phase post *L. amazonensis* inoculation.

#### Distinct transcriptional signatures that could contribute to unique communication with T lymphocytes

DLs represent the most potent source of co-signaling molecules active on naive as well as on T natural regulatory T lymphocytes (Treg). In peripheral tissues, once they have sensed microbial or endogenously derived agonists, DLs generally migrate to the T cell area in DLNs. The distinct values of live amastigote population size observed in the ear pinna of C57BL/6 and DBA/2 mice from days 4 to 22 might reflect distinct priming and or effector/regulatory functions of CD3 T lymphocytes. Indeed, while a pronounced up regulation of transcripts encoding for CD4 and CD8β at day 4 PI (Table 1) was detected in the amastigote-hosting DBA/2 ears, no modulation of these transcripts was detected in amastigote-hosting C57BL/6 ears. We further considered the above features by focusing on any signalling molecules-encoding transcripts otherwise known to act on naive T lymphocytes that cross the HEV of the ear- DLN and migrate through the secondary lymphoid organ matrix. IL-7 (+1.69) and IL-7Rα (+1.58)-encoding transcripts were found positively modulated in DBA/2 DLs. Since DBA/2 DL survival could also be favoured by CXCR4 engagement (+2.47) [47], they could support, through IL-7 and MHC Class II co-expression, the proliferation of both i) CD4+ T lymphocytes [48], [49], [50], [51] and ii) CD8+ T lymphocytes [52]. Interestingly, in ears from DBA/2 mice, natural Treg transcripts were detected at day 4 PI (Table 1; *foxp3* +6.7+/−4.8), a feature that could reflect communication with live amastigotes-hosting DLs: indeed not only the *cd200r3* transcript encoding for a receptor shown to be expressed on regulatory DLs was not down regulated [53] but transcripts encoding for chemokines otherwise known to recruit Tregs (*ccl17* +1.88; RT-qPCR +1.34) were up-regulated. Altogether, in DBA/2 ears, from day 4 to day 22, the parasite load increase could assess the unique communication between regulatory DL and regulatory T lymphocytes. By contrast in ears of C57BL/6 mice, not only no up regulation of *foxp3* transcripts was evidenced at day 4 PI but alternatively two interesting modulations were documented in BMD-DLs i) the down regulation of *cd200r3* transcripts (−2.04) and ii) the up regulation of transcripts encoding for TNFsf4 (OX40L; +1.66; RT-qPCR 7.34 ), a factor shown to inhibit the induction of iTReg cells [54]. The down modulation of *dc-sign* transcripts (*cd209c*: −2.21) could also result in abortive DL-T cell interactions, the binding of DL DC-SIGN to lymphocyte ICAM-3 being impeded [55]. Together with the up-modulation of *tnfrsf23* transcripts (+1.94) and down modulation of *icosl* (*cd275*; −1.56) transcripts, the DC-SIGN regulation could prevent T lymphocytes to be properly activated, thus limiting their proliferation and the developmental process completion to end-stage lymphocytes displaying either effector or regulatory functions [56], [57].

**Table 1:** Monitoring of transcript abundance in the C57BL/6 and DBA/2 ear pinnas collected at day 4 and 15 post inoculation/pi of *L. amazonensis*.

FC in transcripts-ear pinna-	C57BL/6		DBA/2	
	Day 4	Day 15	Day 4	Day 15
*cd4*	1.18±0.57	0.71±0.49	4.7±2.2	0.66±0.07
*cd8β*	0.34±0.6	1.02±0.56	18.8±2.6	4.64±2.2
*foxp3*	1.24±0.33	0.88±0.13	6.7±4.8	0.35±0.31
*cxcr4*	0.79±0.12	0.43±0.42	3.13±2.2	0.55±0.11

Ten thousand metacyclic promastigotes of *L. amazonensis* were inoculated into ear pinna dermis of C57BL/6 and DBA/2 mice. At days 7 and 15 pi, the ear pinnas were removed and processed for RNA isolation. Transcript abundance was determined by real-time RT-qPCR. Results are indicated as Fold Changes (FC) unexposed ears being used for calibrator for the calculation of relative expressions. One representative out of 3 independent experiments with three ears that were individually processed the means and SD being displayed.

In conclusion, the more or less rapid and long term establishment of parasites such as *L. amazonensis* amastigotes, otherwise known to strictly rely on subversion of macrophage and dendritic leucocyte lineages, is expected to reflect stepwise and complex processes taking place in both i) the skin dermis where were inoculated their flagellated ascendants-the metacyclic promastigotes- ii) and the skin-DLN. Relying on mice of two distinct inbred strains- C57BL/6 and DBA/2- that rapidly and durably display distinct phenotypes at the two sites of establishment of *L. amazonensis* amastigotes, we were curious to address the following question: could live *L. amazonensis* amastigotes-hosting DL display unique signatures which account for the distinct phenotypes?

**Figure 6:**
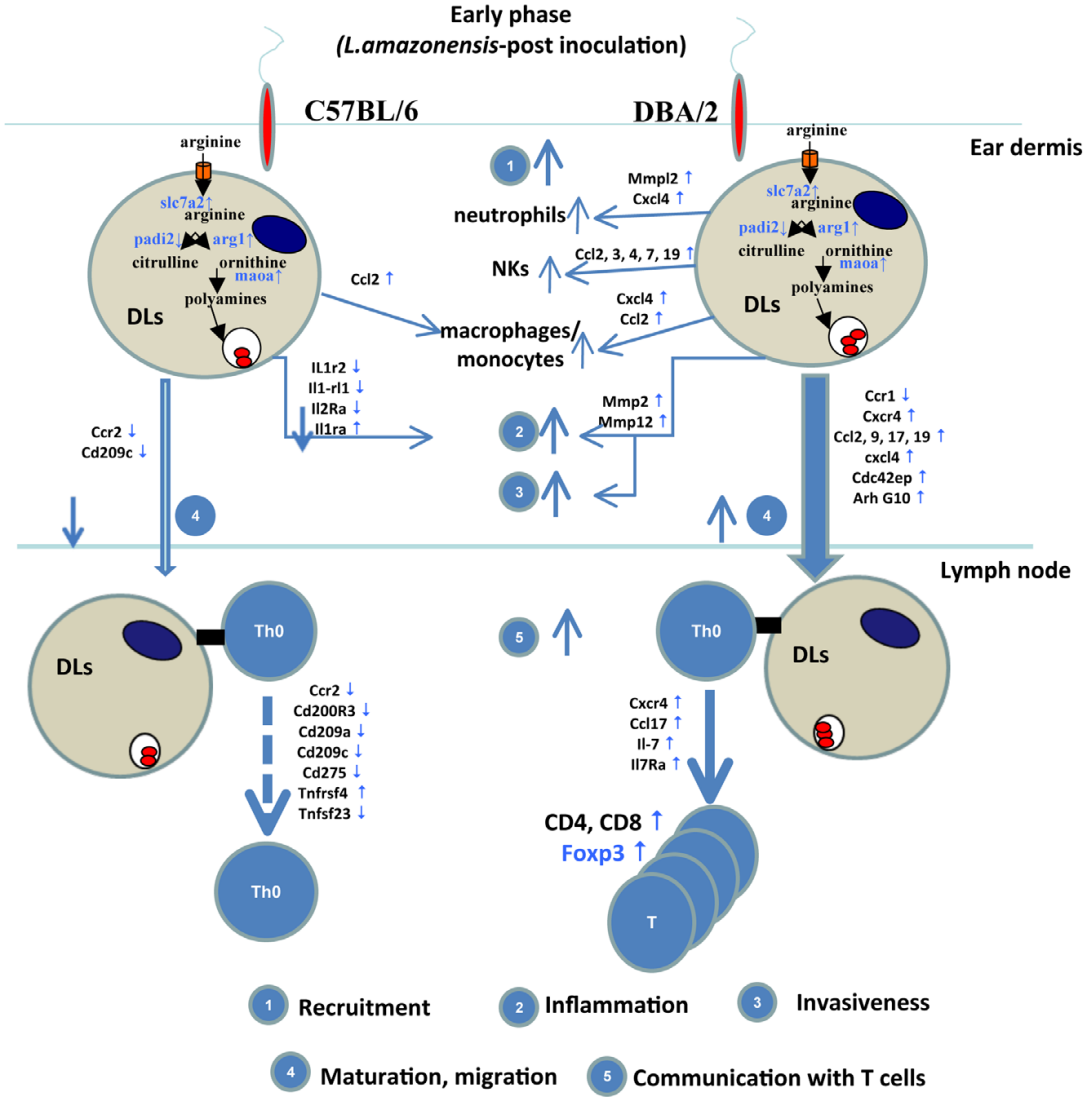
The distinct transcriptional signatures of BMD-DLs housing live LV79 amastigotes. Authors' interpretation of key distinct early events is depicted. Are highlighted five immune processes including DL property to recruit leucocytes and initiate inflammatory processes DL invasiveness (1, 2) and migration (3, 4) DL property to communicate with T lymphocytes (5).

Whatever the mouse genotype origin of the BMD-DLs, the *L. amazonensis* amastigotes establish themselves similarly a phenotype that reflects similar subversion of the DL arginine-dependent pathway. Thus, comparing the other transcriptional profiles as well as the expression of some key proteins allowed highlighting a promising set of transcripts that could account for a more rapid onset of the amastigote population expansion in the C57BL/6 than in the DBA/2 mouse ear pinna post the intra-dermal inoculation of 10^4^ *L. amazonensis*. Our results did evidence that once subverted as cells hosting live *L. amazonensis* amastigotes, DLs from C57BL/6 or DBA/2 differently expressed transcripts involved in recruitment of other non-dendritic leukocytes as well as in the remodelling of the dermis extracellular matrix (Figure 6). The early invasiveness-related phenotype displayed by DBA/2 BMD-DLs could account for the early onset of the amastigote population expansion in the ear pinna. Moreover, the up regulation of the genes encoding chemokines and chemokine receptors suggested that DBA/2 BMD-DLs would be more responsive to chemoattractant gradients and thus amenable to enter into afferent lymphatics.

Whether GM-CSF-dependant-CD11c positive DBA/2 DLs could be the key cells which shuttle the *Leishmania* amastigotes from the ear to the DLN and for delivering signals to regulatory T cells are the DL emigrating from the dermis remains an open question; ; but it is likely that once the Treg are populating the dermis, both live amastigotes-hosting DL and live amastigotes-hosting macrophages cooperate, the outcome being the increaseand the long-term persistence of parasites.Interestingly, the early homing of nTreg in the ear pinna did not prevent the priming and differentiation of T lymphocytes that rapidly control the amastigote population size observed in DBA/2 mice. By contrast, once GM-CSF-dependant CD11c positive C57BL/6 DLs are hosting live *L. amazonensis* amastigotes they do not display any transcriptional signatures that could account for any delivery of signals to T lymphocytes whatever their subsets.

Though we did/do not ignore the limitations of *in vitro* generated DLs, we were expecting that once, sorted on the basis of their status of phagocytic leukocytes hosting live *L. amazonensis* amastigotes, and depending from their mouse genotype origin, these DLs will allow extracting unique signatures that dictate the balance between pro-inflammatory and counter-inflammatory agonists. The latter would operate, within the skin and the skin- DLN, to both i) prevent progressive and irreversible skin damages and ii) promote the rapid skin remodelling as a niche where *L. amazonensis* amastigotes will reach the developmental stage that allows their persistence. Whatever the DL subsets either present or recruited in the dermis, such a rapid reprogramming of *L. amazonensis* amastigotes-hosting DLs is expected to operate: indeed the live amastigotes-derived agonists detected by DL sensors are likely the agonists that dominate over all the other endogenously agonists that allow delineating the distinct lineage subsets that populate the dermis. Thus despite the limitations highlighted above, once any discrete sub-phenotypes have been delineated with quantitative parameters, high content transcriptional profiles emerging from *in vitro* generated populations of DL hosting live *L. amazonensis* amastigotes become solid resources for further biologically sound *in vivo* investigations.

## Supporting Information

Figure S1 **Characterization by FCM and fluorescence microscopy of BMD-DLs from C57BL/6 and DBA/2 mice.** (**A**) Biparametric CD11cPE//MHC class II PE-CY5 dot plot of DBA/2 DL populations sampled 24 hours post the addition or not of live amasitgotes (**B**) Epifluorecence microscopy of CD11c positive leukocyte populations . Surface and intracellular MHC class II molecules were stained by a monoclonal Ab conjugated to PE-CY5 (in red) and cell nuclei were stained by Hoechst 33342 (in blue). Different CD11c positive-cell subsets were evidenced and their percentages displayed in panel (**C**). The red gate in panel (**A**) corresponds to the CD11c^+^ DLs, *i.e.* cells that do express MHC class II molecules at low or high level. (**B**) Cell in upper panel corresponds to a MHC class II^high^ semi-mature-like DL and the one in medium panel is representative of a MHC class II^low^ immature- like DL/iDL. The lower panel shows a representative MHC class II^neg^ Contaminating Cell/CC. Whatever the C57BL/6 or DBA/2 mouse genotype, no significant difference in the percentages of the three cell subsets among the CD11c leukocyte populations generated *in vitro* (C). Median and standard deviations are shown (n = 5 independent experiments).
(TIF)

Figure S2 **Modulation of arginine metabolism and polyamine pathways in LV79-housing DLs.** Total RNAs from sorted BMD-DLs obtained from 3 independent experiments were submitted to Affymetrix-based analyses. The fold change values collected from the analyses of either LV79-hosting or control DLs are indicated for C57BL/6 (black text) and DBA/2 mice (grey text).
(TIF)

Figure S3 **Affymetrix analysis of modulated transcripts in C57BL/6 and DBA-2 GM-CSF responsive BMD–DLs hosting live** ***L. amazonensis*** **amastigotes.** *Ds*Red2-LV79 amastigotes were added or not to cultures of C57BL/6 and DBA/2-DLs. Twenty four hours later, three samples collected from three distinct cultures of either unexposed DLs or live amastigote-hosting DLs were carefully sorted, their total RNA extracted and further processed for Affymetrix-based analyses. To select significant differential gene expression between sorted *L. amazonensis* housing-DLs and unexposed DLs for each mouse genotype a p-value threshold of 0.05 was used. All sample values including standard errors were deposited into GEO database (see Methods).
(DOCX)
