# Supplementary figures and images for: Distinct Transcriptional Signatures of Bone Marrow-Derived C57BL/6 and DBA/2 Dendritic Leucocytes Hosting Live Leishmania amazonensis Amastigotes
Source: PLoS Negl Trop Dis. 2012 Dec 13;6(12):e1980. doi: 10.1371/journal.pntd.0001980 (PMC3521701; doi:10.1371/journal.pntd.0001980)

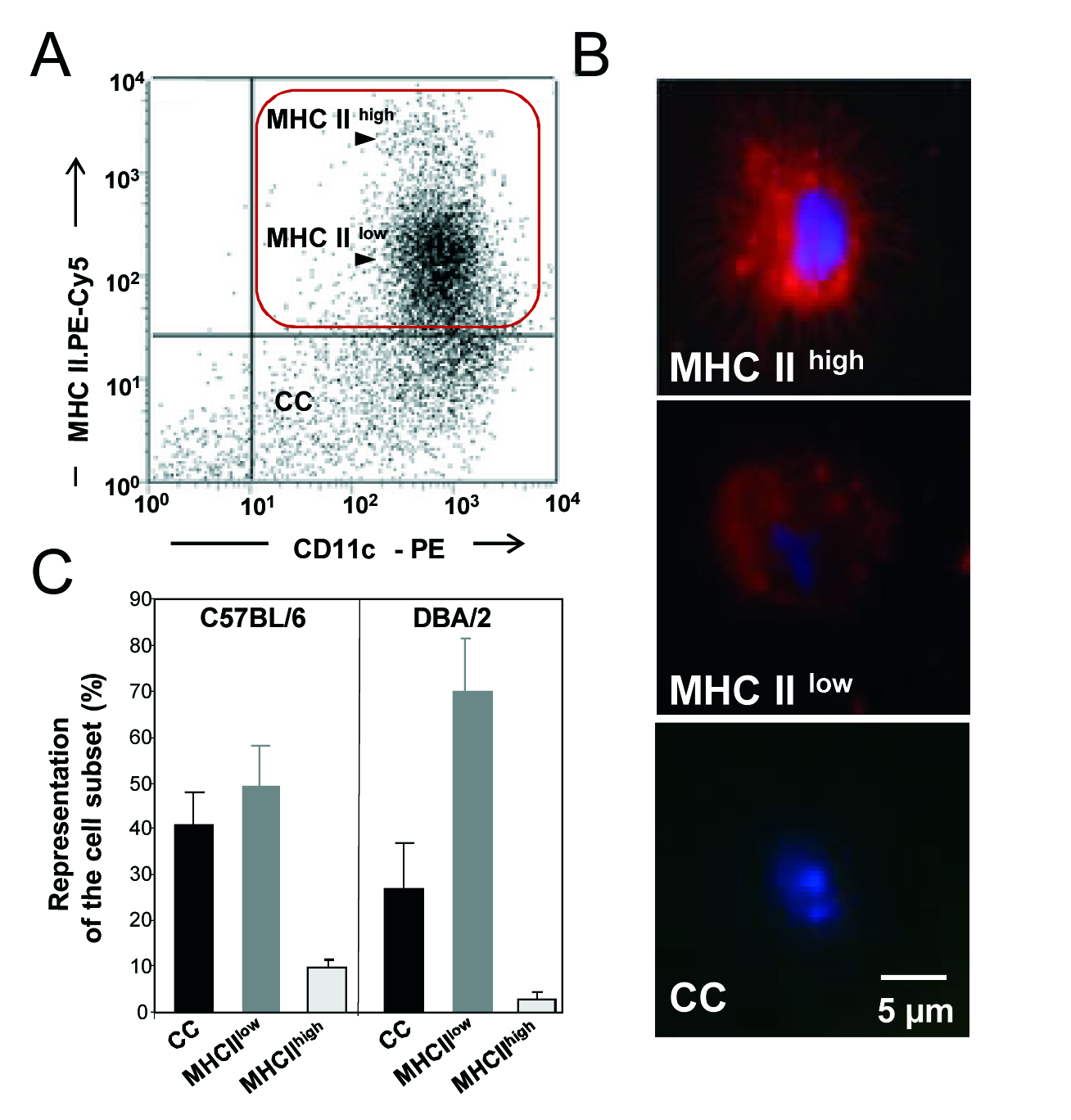

Supplement: Figure S1 — Characterization by FCM and fluorescence microscopy of BMD-DLs from C57BL/6 and DBA/2 mice. (A) Biparametric CD11cPE//MHC class II PE-CY5 dot plot of DBA/2 DL populations sampled 24 hours post the addition or not of live amasitgotes (B) Epifluorecence microscopy of CD11c positive leukocyte populations . Surface and intracellular MHC class II molecules were stained by a monoclonal Ab conjugated to PE-CY5 (in red) and cell nuclei were stained by Hoechst 33342 (in blue). Different CD11c positive-cell subsets were evidenced and their percentages displayed in panel (C). The red gate in panel (A) corresponds to the CD11c+ DLs, i.e. cells that do express MHC class II molecules at low or high level. (B) Cell in upper panel corresponds to a MHC class IIhigh semi-mature-like DL and the one in medium panel is representative of a MHC class IIlow immature- like DL/iDL. The lower panel shows a representative MHC class IIneg Contaminating Cell/CC. Whatever the C57BL/6 or DBA/2 mouse genotype, no significant difference in the percentages of the three cell subsets among the CD11c leukocyte populations generated in vitro (C). Median and standard deviations are shown (n = 5 independent experiments). (TIF) [file pntd.0001980.s001.tif]

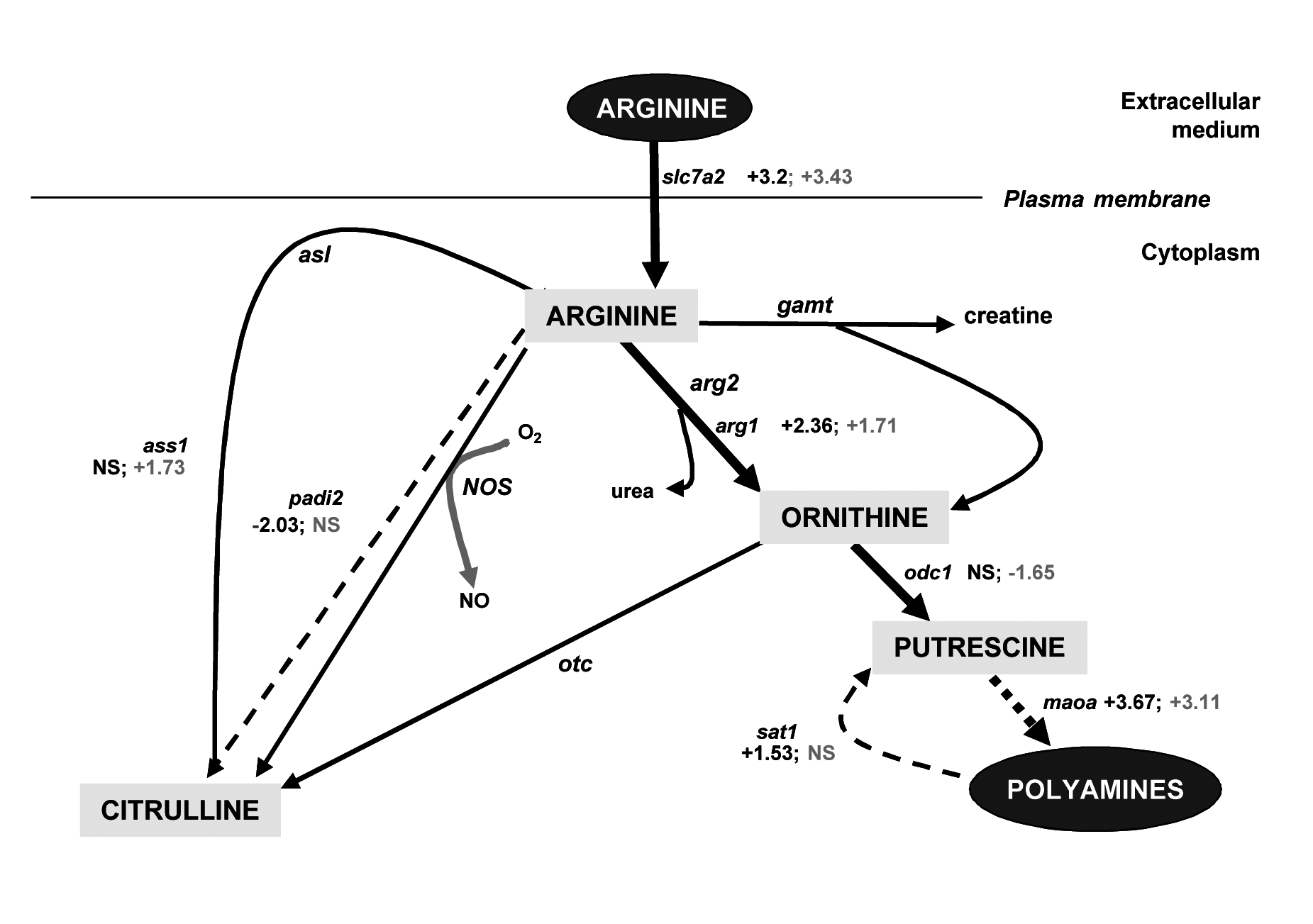

Supplement: Figure S2 — Modulation of arginine metabolism and polyamine pathways in LV79-housing DLs. Total RNAs from sorted BMD-DLs obtained from 3 independent experiments were submitted to Affymetrix-based analyses. The fold change values collected from the analyses of either LV79-hosting or control DLs are indicated for C57BL/6 (black text) and DBA/2 mice (grey text). (TIF) [file pntd.0001980.s002.tif]
