# Supplementary material for: Distinct Transcriptional Signatures of Bone Marrow-Derived C57BL/6 and DBA/2 Dendritic Leucocytes Hosting Live Leishmania amazonensis Amastigotes
Source: PLoS Negl Trop Dis. 2012 Dec 13;6(12):e1980. doi: 10.1371/journal.pntd.0001980 (PMC3521701; doi:10.1371/journal.pntd.0001980)
Supplement: Figure S3 — Affymetrix analysis of modulated transcripts in C57BL/6 and DBA-2 GM-CSF responsive BMD–DLs hosting live L. amazonensis amastigotes. DsRed2-LV79 amastigotes were added or not to cultures of C57BL/6 and DBA/2-DLs. Twenty four hours later, three samples collected from three distinct cultures of either unexposed DLs or live amastigote-hosting DLs were carefully sorted, their total RNA extracted and further processed for Affymetrix-based analyses. To select significant differential gene expression between sorted L. amazonensis housing-DLs and unexposed DLs for each mouse genotype a p-value threshold of 0.05 was used. All sample values including standard errors were deposited into GEO database (see Methods). (DOCX) [file pntd.0001980.s003.docx]

**Supporting data S3**

| **Abbreviation** | **Name** | T**ranscript cluster ID** | **Fold Change Affymetrix** | | **p value** | |
| --- | --- | --- | --- | --- | --- | --- |
|  |  |  | **C57BL/6** | **DBA-2** | **C57BL/6** | **DBA-2** |
| ***arg1*** | arginase 1 | 10368343 | **+2.36** | **+1.71** | **6.57E-04** | **3.06E-02** |
| ***arhap10***  ***ass1*** | Rho GTPase activating protein 10    argininosuccinate synthetase 1 | 10579776  10471154 | **/**  **/** | **+2.15**  **+1.73** | **NS**  **NS** | **2.19E-03**  **3.30E-03** |
| ***ccl2*** | chemokine (C-C motif) Ligand 2 | 10379511 | **+1.90** | **+3.38** | **2.26E-03** | **4.23E-10** |
| ***ccl3*** | chemokine (C-C motif) Ligand 3 | 10389231 | **+3.61** | **+4.58** | **<1.00E-13** | **<1.00E-17** |
| ***ccl4*** | chemokine (C-C motif) Ligand 4 | 10379721 | **/** | **+2.58** | **NS** | **3.76E-06** |
| ***ccl6*** | chemokine (C-C motif) Ligand 6 | 10389222 | **/** | **+1.69** | **NS** | **9.19E-07** |
| ***ccl7*** | chemokine (C-C motif) Ligand 7 | 10379518 | **/** | **+2.57** | **NS** | **1.01E-05** |
| ***ccl9*** | chemokine (C-C motif) Ligand 9 | 10392839 | **+1.46** | **+2.13** | **4.89E-02** | **2.37E-03** |
| ***ccl17*** | chemokine (C-C motif) Ligand 17 | 10574226 | **/** | **+1.88** | **NS** | **3.20E-05** |
| ***ccl19*** | chemokine (C-C motif) Ligand 19 | 10512322 | **/** | **+1.16** | **NS** | **2.80E-02** |
| ***ccr1*** | chemokine (C-C motif) Receptor 1 | 10598004 | **/** | **-1.68** | **NS** | **7.43E-03** |
| ***ccr2*** | chemokine (C-C motif) Receptor 2 | 10590631 | **-1.68** | **/** | **3.16E-03** | **NS** |
| ***Cdc42ep***  ***cd200*** | CDC42 effector protein (Rho GTPase binding) 2  cd200 antigen | 10465278  10439651 | /  **+1.99** | **+1.64**  **+2.74** | **NS**  **1.20E-03** | **4.42E-02**  **2.29E-02** |
| ***cd200r3*** | cd200 receptor 3 | 10435937 | **-2.04** | **/** | **1.39E-02** | **NS** |
| ***cd200r4*** | cd200 Receptor 4 | 10435920 | **/** | **+1.63** | **NS** | **2.29E-02** |
| ***cd205*** | ly75 lymphocyte antigen 75 | 10482929 | **+1.76** | **+1.81** | **3.90E-05** | **2.50E-02** |
| ***cd209c*** | (dc-sign) | 10576829 | **-2.21** | **/** | **1.83E-02** | **NS** |
| ***cd274*** | cd274 antigen (B7-H1) | 10462390 | **+1.80** | **+1.83** | **4.59E-03** | **1.38E-02** |
| ***cd300e*** | cd300 antigen | 10392839 | **-2.17** | **-2.00** | **1.63E-03** | **2.19E-02** |
| ***cd86*** | cd86 antigen | 10439312 | **/** | **-1.51** | **NS** | **2.57E-02** |
| ***cxcl4*** | chemokine (C-X-C motif) ligand 4 | 10523134 | **/** | **+2.88** | **NS** | **1.06E-07** |
| ***cxcl14*** | chemokine (C-X-C motif) ligand 14 | 10409579 | **/** | **+2.97** | **NS** | **2.27E-08** |
| ***cxcr4*** | chemokine (C-X-C motif) receptor 4 | 10357472 | **/** | **+2.47** | **NS** | **1.12E-05** |
|  |  |  |  |  |  |  |
| ***icosl*** | icos ligand | 10364361 | **-1.56** | **/** | **1.64E-02** | **NS** |
| ***il1α*** | interleukin 1, alpha | 10487588 | **+3.02** | **+2.99** | **2.17E-06** | **6.74E-05** |
| ***il1f9*** | interleukin 1 family, member 9 | 10469786 | **+3.40** | **+3.82** | **9.28E-06** | **2.61E-13** |
| ***il1r2*** | interleukin 1 receptor type II | 10345752 | **-1.94** | **/** | **8.99E-06** | **NS** |
| ***il1rl1*** | interleukin receptor-like 1 | 10345791 | **-2.38** | **/** | **7.58E-07** | **NS** |
| ***ilrn*** | interleukin 1 receptor antagonist | 10469816 | **+1.69** | **+2.05** | **4.36E-02** | **3.73E-04** |
| ***il2ralpha*** | interleukin 2 receptor, alpha chain | 10469278 | **-1.99** | **/** | **1.68E-07** | **NS** |
| ***il7*** | interleukin 7 | 10497199 | **/** | **+1.69** | **NS** | **3.28E-02** |
| ***il7rα*** | interleukin 7 receptor alpha (CD127) | 10427628 | **/** | **+1.58** | **NS** | **2.12E-02** |
| ***il10rβ*** | interleukin 10 Receptor beta | 10436841 | **/** | **+1.60** | **NS** | **2.79E-02** |
| ***il18rap*** | interleukin 18  receptor accessory protein | 10345824 | **-1.96** | **-1.67** | **3.41E-04** | **4.87E-03** |
| ***il18r1*** | interleukin 18 receptor 1 | 10345807 | **-1.81** | **-1.95** | **1.07E-04** | **1.84E-03** |
| ***maoa***  ***mmp2*** | monoamine oxidase A  matrix metallopeptidase 2 | 10598771  10573924 | **+3.67**  **/** | **+3.11**  **+2.53** | **4.54E-07**  **NS** | **1.8E-08**  **1.04E-07** |
| ***mmp8*** | matrix metallopeptidase 8 | 10583100 | **+1.87** | **+2.53** | **9.28E-06** | **4.82E-08** |
| ***mmp12*** | matrix metallopeptidase 12 | 10583056 | **/** | **+1.45** | **NS** | **1.76E-02** |
| ***mmp13*** | matrix metallopeptidase 13 | 10583044 | **+2.40** | **+3.66** | **2.74E-03** | **2.55E-10** |
| ***mmp14*** | matrix metallopeptidase 14 | 10415052 | **+2.04** | **+2.36** | **7.53E-04** | **4.19E-06** |
| ***mmp19*** | matrix metallopeptidase 19 | 10367400 | **+2.78** | **+2.04** | **1.65E-08** | **1.60E-06** |
| ***odc1*** | ornithine decarboxylase, structural 1 | 10394770 | / | **-1.65** | **NS** | **7.12E-03** |
| ***padi2*** | peptidyl arginine deiminase, type II | 10509838 | **-2.03** | **/** | **2.52E-05** | **NS** |
| ***sat1*** | spermidine/spermine N1-acetyl transferase 1 | 10607467 | **+1.53** | **/** | **1.62E-02** | **NS** |
| ***slc7a2*** | solute carrier family 7, member 2 | 10571444 | **+3.20** | **+3.43** | **1.65E-09** | **2.61E-13** |
| ***timp2*** | tissue inhibitor of metalloproteinase 2 | 10393559 | **+1.93** | **+3.22** | **9.05E-04** | **4.47E-08** |
| ***tnfoip813*** | tumor necrosis factor, alpha-induced protein 8-like 3 | 10593646 | **-1.83** | **/** | **7.53E-04** | **NS** |
| ***tnfsf4*** | Tumor necrosis factor (ligand) superfamily, member 4 (OX40L) | 10351095 | **+ 1.66** | **/** | **2.84E-04** | **NS** |
| ***tnfsf8*** | tumor necrosis factor (ligand) superfamily, member 8 | 10513729 | **-2.18** | **-1.79** | **4.58E-04** | **1.72E-02** |
| ***tnfsf26*** | tumor necrosis factor (ligand) superfamily, member 26 | 10569485 | **+2.92** | **+3.05** | **6.59E-08** | **6.23E-08** |
| ***tnfrsf1b*** | tumor necrosis factor receptor superfamilly member 1b | 10518300 | **+1.64** | **+2.24** | **1.60E-04** | **3.86E-03** |
| ***tnfrsf21*** | tumor necrosis factor receptor superfamilly member 21 (DR6) | 10445241 | **+1.53** | **/** | **1.22E-03** | **NS** |
| ***tnfrsf23*** | tumor necrosis factor receptor superfamilly member 23 (TNFRh1) | 10569504 | **+ 1.94** | **/** | **4.90E-03** | **NS** |
